# Predictors for functional and anatomic outcomes in macular edema secondary to non-infectious uveitis

**DOI:** 10.1371/journal.pone.0210799

**Published:** 2019-01-24

**Authors:** Jessica Matas, Victor Llorenç, Alex Fonollosa, Cristina Esquinas, David Diaz-Valle, Barbara Berasategui, Marina Mesquida, Joseba Artaraz, Jose Rios, Alfredo Adan

**Affiliations:** 1 Clinic Institute of Ophthalmology (ICOF), Hospital Clinic of Barcelona, Barcelona, Spain; 2 August Pi i Sunyer Biomedical Research Institute (IDIBAPS), Barcelona, Spain; 3 Department of Ophthalmology, BioCruces Health Research Institute, Hospital Cruces, University of the Basque Country, Baracaldo, Spain; 4 Valle Hebron Research Institute, Autonomous University of Barcelona, Barcelona, Spain; 5 Ophthalmology Department and Health Research Institute (IdISSC), Hospital Clinic of San Carlos, Madrid, Spain; 6 Medical Statistics Core Facility, IDIBAPS, Barcelona, Spain; Oregon Health and Science University, UNITED STATES

## Abstract

**Aims:**

We aimed to investigate predictive factors for visual and anatomic outcomes in patients with macular edema secondary to non-infectious uveitis.

**Material and methods:**

We conducted a multicenter, prospective, observational, 12-month follow-up study. Participants included in the study were adults with non-infectious uveitic macular edema (UME), defined as central subfoveal thickness (CST) of >300 μm as measured by spectral domain optical coherence tomography (SD-OCT) and fluid in the macula. Demographic, clinical and tomographic data was recorded at baseline, 1, 3, 6 and 12 months. Foveal-centered SD-OCT exploration was set as the gold-standard determination of UME using a standard Macular Cube 512x128 A-scan, within a 6 x 6 mm^2^ area, and the Enhanced High Definition Single-Line Raster. To assess favorable prognosis, the main outcomes analyzed were the best-corrected visual acuity (BCVA) and the CST. Favorable prognosis was defined as sustained improvement of BCVA (2 lines of gain of the Snellen scale) and CST (decrease of 20% of the initial value or <300 μm) within a 12 month period.

**Results:**

Fifty-six eyes were analyzed. The number of eyes with sustained improvement in the CST was 48 (86.2%), against 23 (41.1%) eyes with sustained improvement in BCVA. Favorable prognosis, as defined above, was observed in 18 (32.1%) eyes. UME prognosis was negatively correlated with baseline foveal thickening, alteration in the vitreo-macular interface and cystoid macular edema. In contrast, bilaterally, systemic disease and the presence of anterior chamber cells were predictive of favorable prognosis.

**Conclusion:**

Available treatment modalities in UME may avoid chronic UME and improve anatomic outcome. However, the proportion of functional amelioration observed during 12 months of follow-up is lower. Thicker CST, alteration in the vitreo-macular interface and cystoid macular edema may denote less favorable prognosis. Conversely, bilaterally, systemic disease and anterior chamber cells may be associated with favorable prognosis in UME.

## Introduction

Macular edema (ME) is the main cause of visual impairment and the most frequent structural ocular complication in patients with uveitis [1–3].

Uveitic macular edema (UME) may persist and lead to visual acuity (VA) loss even with adequate control of uveitis activity [4–5].

Currently the specific mechanisms that contribute to the pathogenesis of inflammatory ME are not well defined. It remains unclear why some patients have a single episode, whereas others develop recurrent or chronic UME [6].

The initial factors involved in UME include toxic effects on the retinal vessels and retinal pigment epithelium, inflammatory mediators in the eye and mechanical tractions. There is therefore a breakdown of the blood-retinal barrier and ME develops when the leakage of fluid across the retina vessel wall and through retinal pigment epithelium accumulates within the macular area [7].

Previously ME was assessed by measuring the area of macular leakage with fluorescein angiography (FA) [8]. More recently however, optical coherence tomography (OCT) has been engineered to perform similar functions as FA by measuring retinal thickness [9–10]. OCT has now replaced FA for controlling ME, in part because VA is associated more with retinal thickness than with macular leakage [9–10].

New local and systemic therapies, such as intravitreal dexamethasone slow-release implants and biologic drugs, have been approved for treatment of uveitis in recent years. Information about the functional effect of these therapies and the anatomical location of their action however remains unclear.

Little data exists regarding the factors influencing sustained anatomic and visual recovery in eyes with UME, whilst most of studies performed are still retrospective. To attempt to gauge this gap in current knowledge we performed a prospective study with uveitis to determine if demographic, clinical features, as well as specific OCT findings, could predict a favorable prognosis in eyes with UME.

## Material and methods

### Study design

We performed a multicenter, prospective, observational, 12-month follow-up study.

All patients with UME in at least one eye were proposed for inclusion. Three referral centers for ocular inflammatory diseases in Spain (Hospital Clinic of Barcelona, Hospital Cruces and Hospital Clínico San Carlos) participated in the recruitment of patients from January 2015 to January 2017.

### Inclusion and exclusion criteria

Adult patients with non-infectious uveitis who presented with ME in at least one eye were included if the following criteria were met: 1) BCVA of 20/60 or less but greater than 20/200 2) CST of >300 μm as measured by SD-OCT (Cirrus, Carl Zeiss Meditec, Dublin, CA) and fluid in the macula. Exclusion criteria were limited to infectious uveitis, unclear media (confidentiality analysis <5), concomitant ocular diseases that compromise visual prognosis independently of ME, pregnancy, immunocompromising systemic diseases (including, but not limited to AIDS, leukemia, lymphoma, chemotherapy etc.) and eyes with any intervention (surgery, intravitreal therapy) in the previous 4 months.

Only one eye from each study participant was enrolled. Whenever bilateral UME was presented and both eyes met inclusion criteria, the eye with the thickest UME was selected. The Standardization of Uveitis Nomenclature Working Group criteria was used to anatomically classify the uveitis[11].

### Ethics statement

Ethical and legal restrictions did apply according to the IRB statement on the project’s approval (current Spanish law on Data Protection: Organic Law 15/1999). Local ethics committees approved the study (Comité ético de Investigación Clínica del Hospital Clínic de Barcelona 2013/8574; Comité de ética de la investigación con medicamentos de Euskadi, Hospital Universitario Cruces PI201406; Comité ético de investigación clínica del Hospital Clínico San Carlos de Madrid 13/ 244-E). All patients provided written informed consent, and the research followed the regulations of the Declaration of Helsinki.

### Ophthalmologic assessment

The following mandatory protocol-based assessments were performed and were reported in the present study: at baseline, and at 1, 3, 6 and 12 months. Other visits at different time-points were allowed, at the discretion of the treating physician.

These visits were conducted at each clinical site by the investigators and data was recorded in an electronic case report form designed *ad hoc*. Ancillary tests were ordered at the investigator discretion.

During each appointment, a complete eye examination was carried out, including best corrected visual acuity (BCVA), slit lamp examination, Goldman applanation tonometry, indirect fundus examination under pupil dilation and SD-OCT. BCVA was performed with Snellen charts in decimal scale at a test distance of 6 m. Results on BCVA were converted to logarithm of the minimum angle of resolution for statistical analysis. Inflammatory activity, that is the presence or absence of anterior chamber cells, vitreous haze or posterior segment inflammatory signs as judged by the investigator, was recorded at each visit. Other imaging methods, e.g., FA, were optional and were left to the discretion of the researcher. Although FA was not included as protocol-based assessment in our study, it was indicated specifically to evaluate physiologic macular leakage and uveitis (e.g., for suspected vasculitis, neovascularization, optic nerve leakage…).

At each visit a standardized questionnaire about past and current ophthalmic events and treatments was recorded. Treatments were divided into local (peri/intraocular injection of triamcinolone or intravitreal dexamethasone implant), systemic (oral corticosteroids, immunosuppressive or biologic drugs) or a combination of both. Although there are no guidelines for the treatment of UME, it was recommended to treat UME until a resolution, if there was no contraindication. The decision on the treatment for local and/or systemic therapy was influenced by the severity of the inflammation and the UME, and by uni- or bilaterally of the uveitis and UME. Our first concern in the treatment of UME was to diminish inflammatory activity and maintain or improve BCVA. Even eyes with long-standing ME with structural changes and macular atrophy were treated as needed in order to achieve ME disappearance. Eyes could receive more than one injection for their UME if the investigator determined it to be medically necessary. Eyes with no BCVA improvement or those with no CST reduction after intravitreal corticosteroids were considered non-responding to the treatment and could be not retreated at the investigator’s discretion. Pars plana vitrectomy was limited to remove inflammatory mediators in the vitreous and vitreo-macular traction/epiretinal membrane.

### SD-OCT

All subjects underwent SD-OCT exploration as gold-standard determination of UME using Macular cube 512x128 A-scan, within a 6 x 6 mm^2^ area centered on the fovea; and the Enhanced High Definition Single-Line Raster, which collected data along a 6 mm horizontal line. Imaging assessment was performed by two masked investigators who were blind to clinical data of the corresponding patients. In the event of discrepancies, the two graders made the assessment together and reached a consensus. Masked investigators were asked to determine the presence of UME and the qualitative and quantitative SD-OCT findings.

The Macular cube 512x128 A-scan was used to assess the pattern of UME (sub-retinal fluid, cystoid, diffuse or tractional ME), the CST, the macular volume and the diameter of the greatest cyst if present. Foveal thickness between the Bruch’s membrane and the inner limiting membrane within the central Early Treatment Diabetic Retinopathy Study subfield (foveal thickness within 500 μm of the foveal center) defined CST. Macular edema was defined as CST of > 300 μm as measured by SD-OCT and fluid in the macula. Any cystic space was considered cystoid macular edema (CME) if ME met the above mentioned definition. The Enhanced High Definition Single-Line Raster scan was used to assess the disruption of the ellipsoid line, the vitreo-macular interface (VMI), the sub-retinal fluid (SRF) and the subfoveal choroidal thickness. Subfoveal choroidal thickness was measured at the fovea from the posterior edge of the retinal pigment epithelium to the choroid/sclera junction.

### Outcome measures

The main outcome measures analyzed were BCVA and CST. Sustained visual improvement was defined as 2 lines of maintained gain of the Snellen BCVA (-0.2 base 10 logarithm of BCVA decimal fraction–equivalent or a more favorable change in BCVA) at 12 months of follow-up. Otherwise, improvement of CST was defined as a maintained decrease of 20% of the baseline value or reduction to less than 300 μm at 12 months of follow-up. To define favorable prognosis of eyes with UME, we focused on eyes with improvement in BCVA and CST, as per the above mentioned definitions, who presented sustainment of both outcome measures within a 12 month period. Conversely, eyes with non-favorable prognosis were those who did not improve both outcomes at 12 months of follow-up.

### Statistical analysis

To describe the qualitative variables, absolute frequencies and percentages were used. The description of quantitative variables was performed using the mean and standard deviation (SD). The Kolmogorov-Smirnov test was used to assess the normality of distributions.

In the case of quantitative variables, the comparison of the characteristics of the eyes depending on the presence of favorable prognosis, BCVA and CST, was carried out using the Student t-test (or Mann-Whitney U-test if normality was not assumed). The Chi-squared test (Fisher test for frequencies <5) was used for the comparison of categorical variables. Wilcoxon Test was performed in order to analyze changes in the main outcomes.

A final model was developed using back stepwise logistic regression analysis including favorable prognosis as the dependent variable. Variables with a significance <0.1 in the univariate analysis were included as independent variables. The results have been described with odds ratio (OR) with a 95% confidence interval (CI) and *p*-values. The Hosmer-Lemeshow goodness-of-fit test was performed to assess the overall fit of the model [12]. The combination of predictors from the final models was used to calculate the probabilities of favorable prognosis. The probability of favorable prognosis in this population was calculated by the following formula: *Exp (b) / (1 + Exp (b))*, where b = -2.933 + 2.100 (in the case of laterality) + 2.367 (in the case of anterior chamber cells). Finally, Receiver Operating Characteristic curve analysis was used to assess the predictive power to the favorable prognosis. For all the tests *p*-values <0.05 were considered statistically significant. The statistical package R Studio (version 2.5) was used for the statistical analyses.

## Results

### Baseline characteristics and outcomes

Fifty-six eyes from fifty-six patients with UME were included in the study. The mean age of patients was 51 years (range 21–89 years) and the male-to-female ratio was 3:4. Most patients were Caucasian (n = 54). The mean time from the first episode of UME to baseline was 18.3 months (range 0–144 months). From analyzing anatomic sites of inflammation, posterior uveitis was the predominant anatomical diagnosis (n = 20, 36% of eyes); 15 (26%) eyes presented with anterior uveitis, 12 (22%) with panuveitis and 9 (16%) with intermediate uveitis. The most frequent systemic disorders associated with uveitis were seronegative spondyloarthropathies, Vogt-Koyanagi-Harada syndrome and sarcoidosis, whereas Birdshot chorioretinopathy was the main ocular syndrome. Macular edema occurred in unclassified uveitis in 13 (23.2%) eyes (Table 1).

**Table 1:** Baseline characteristics at the time of UME diagnosis.

Baseline Characteristics	Eyes, n (%)
**Age** (years, mean, range)	51 (21–89)
**Gender**	
Male	24 (42.9)
Female	32 (57.1)
**Race**	
Caucasian	54 (97)
Not Caucasian	2 (3)
**Time from first UME episode to baseline** (months, mean, range)	18.34 (0–144)
**Chronic UME**	
UME not chronic (≤6 months)	32 (57.1)
UME chronic (>6 months)	24 (42.9)
**Primary uveitis diagnosis**	
Unilateral	35 (62.5)
Bilateral	21 (37.5)
**Etiological diagnosis**	
Ocular	19 (34)
Systemic	24 (42.8)
Not classified	13 (23.2)
**Anatomical diagnosis**	
Anterior	15 (26)
Intermediate	9 (16)
Posterior	20 (36)
Panuveitis	12 (22)
**Treatment**	
Topical (only)	2 (3.6)
Local (only)	14 (25.5)
TC (peri/intraocular) Dexamethasone implant	5 9
Systemic ± topical/local	38 (67.3)
Oral Corticosteroids Immunosupressive drugs Biologic drugs	35 19 13
**Pars plana vitrectomy**	2 (3.6)
**Patterns of UME**	
CME	43 (76.8)
DRT	12 (21.4)
SRF	21 (38)
Tractional	16 (25)

Abbreviations: UME, uveitic macular edema; TC, triamcinolone; CME, cystoid macular edema; DRT, diffuse retinal thickening; SRF, sub-retinal fluid

Mean BCVA (LogMAR) improved from 0.45 at baseline to 0.33 at 1 month follow-up (p<0.001), and reached the maximum at 3 months (0.24, p<0.001).

Mean CST at baseline was 437.83 μm, but decreased significantly after 1 month to 357.39 μm (p<0.001), followed by a mean sustained improvement, reaching 329.65 μm at 12 months (p<0.001) (Table 2).

**Table 2:** Mean values of the principal outcome measures: BCVA and CST in 56 eyes with UME at baseline and throughout different time-points of follow-up.

Outcomes	Baseline	Month 1	Month 3	Month 6	Month 12
**BCVA** (LogMAR, SD)	0.45 (0.36)	0.33 (0.31)	0.24 (0.25)	0.26 (0.3)	0.3 (0.32)
Change from baseline (LogMAR, SD)		0.13 (0.23)	0.2 (0.28)	0.19 (0.26)	0.14 (0.31)
P-value*		<0.001	<0.001	<0.001	<0.001
P-value**		<0.001	0.038	0.357	0.216
**CST** (μm, SD)	437.83 (122.22)	357.39 (118.89)	341.79 (107.9)	337.73 (135.48)	329.65 (108.13)
Change from baseline (μm, SD)		-82.56 (143.71)	-83.1 (131.71)	-89.22 (153.8)	-96.44 (154.02)
p-value*		<0.001	<0.001	<0.001	<0.001
p-value**			0.012	0.396	0.477

Abbreviations: BCVA, best corrected visual acuity; CST, central subfoveal thickness; UME, uveitic macular edema; SD, standard deviation

* Baseline; Wilcoxon Test.

** Previous time-points; Wilcoxon Test.

In our cohort, after 12 months of follow-up, the number of eyes with sustained improvement of CST was 48 (86.2%), against 23 (41.1%) eyes with sustained visual improvement. However, favorable prognosis, as sustained improvement of both CST and BCVA, was observed in 18 (32.1%) eyes. There was no significant association with favorable prognosis of UME and demographic items including gender and age (p>0.05).

### Clinical factors associated with favorable prognosis

The anatomic pattern of uveitis increased the likelihood of sustained visual improvement, in eyes with only anterior uveitis more readily experiencing sustained gains in vision than those with intermediate, posterior, or panuveitis (p<0.05). CST improvement however did not vary significantly across the anatomic classifications (p = 0.999).

In relation to whether the disease was only ocular or systemic, a higher favorable prognosis was observed in the group with systemic disease (p = 0.004).

No significant association was found between chronic UME and the prognosis. In our cohort, none of the initial treatment modalities, either local, systemic, or a combination of both, were associated with favorable prognosis regarding the short-term and final functional and anatomic outcome (p = 0.273). In relation to whether the UME was unilateral or bilateral, there was a significant difference in cases with bilateral UME since these cases showed favorable prognosis (OR: 3.07; CI 95% 0.96–9.83; p = 0.049). The presence of cells in the anterior chamber at any degree was associated with favorable prognosis (OR: 4.46; CI 95% 1.31–15.16; p = 0.017). No significant association was found between anterior chamber cells and/or vitreous haze and persistent macular edema (p = 0.123). No relationship was found between the prognosis and the clinical variables of cataract (p = 0.928), keratic precipitates (p = 0.595), vitreous haze (p = 0.256) or chorioretinitis (p = 0.546).

In the multivariate analysis, the anterior chamber cells (OR: 10.66; CI 95% 2.01–14.50, P = 0.005) and the bilaterally (OR: 8.17, CI 95% 1.57–22.57; p = 0.012) were independently related to favorable prognosis. A probability model including these two independent clinical variables showed that the probability of favorable prognosis increased with the number of putative predictors. The probability for favorable prognosis without any of these characteristics would therefore be 5.1%, overcoming 82.3% for those having both variables (Table 3).

**Table 3:** Probability of favorable prognosis including bilaterally and anterior chamber cells in UME.

%	Laterality	Anterior chamber cells
5.1	Unilateral	Absent
30.3	Bilateral	Absent
36.2	Unilateral	Present
82.3	Bilateral	Present

Abbreviations: UME, uveitic macular edema

The final model was calibrated with p values of 0.920 by using the Hosmer-Lemeshow test. The capacity of the significant variables derived from the logistic regression model to predict favorable prognosis was evaluated through Receiver Operating Characteristic curve with an area under the curve of 0.760 (CI 95% 0.630–0.900; p<0.001).

### Influence of OCT parameters on favorable prognosis

When the eyes were analyzed according to the type of UME, CME was reported in 43 (76.8%) eyes, SRF noted in 21 (38%) eyes, alteration in the VMI in 16 (25%) and diffuse retinal thickening (DRT) in 12 (21.4%) of the eyes with uveitis (Table 1). It was observed that CME was a predictive factor of less favorable prognosis. Also noted, the bigger the cyst was, the less favorable the prognosis was found to be. No significant difference was observed between other patterns of OCT, as DRT or SRF patterns were not found to be predictors of favorable prognosis in our cohort (p = 0.427 and p = 0.732 respectively). Conversely, the presence of alteration in the VMI was associated with less sustained visual and CST improvement throughout the follow-up (OR: 9.23; CI 95% 1.35–35.28; p = 0.026). Eyes without alteration of the VMI showed a significantly higher sustained visual improvement (p = 0.016).

Eyes with alteration in the ellipsoid layer at baseline had significantly higher sustained improvement of the CST during the follow-up (p = 0.016), though no association was found between the BCVA and the ellipsoid layer at baseline and throughout the follow-up (p = 0.135).

Finally, no significant association was observed between macular volume measures and favorable prognosis (OR: 0.80); CI 95% 0.57–1.12; p = 0.193) nor between choroidal thickness values and favorable prognosis (p = 0.223).

## Discussion

There is a clear gap in scientific literature investigating the long-term prognosis and the efficacy of several treatment modalities and little is known about clinical and structural findings that can influence UME response. This present study provides data on the functional and anatomical status of eyes with UME during 12 months of follow-up, investigating clinical and tomographic variables influencing patient outcome, regardless of the therapeutic approach used.

Interestingly, results revealed that the proportion of improvement in BCVA and CST obtained in our cohort peaked at 3 months, potentially suggesting that a substantial number of eyes with UME present an important visual recovery upon receiving attention. Three months after receiving treatment it seemed that VA and CST were similar to the vision and the CST maintained throughout the follow-up period.

In our study, in order to determine the prognosis of UME, the sustainment of the improvement of BCVA and CST was taken into account as a whole. If VA and CST were analyzed separately, it was observed that the rate of sustained improvement was higher for CST. High values of CST at baseline negatively affected the final visual and anatomic prognosis, but no relation was found between the value of the initial VA and the final prognosis. This finding can be explained by other factors that could influence VA at baseline, such as outer retina integrity, macular perfusion, etc. Our study is in agreement with other authors who reported a negative correlation between foveal thickness and VA in ME [13–14]. The CST calculation is therefore important for a functional prognostic assessment; however, in our experience, the macular volume seems less valuable than the CST because it correlates with BCVA with no significant difference. Other recent publications using SD-OCT in diabetic ME also showed a correlation between the total macular volume and VA without significant difference [15–16].

Neither gender nor age were found to significantly alter the course of visual improvement. With respect to age, we could not find differences in sustained visual or CST improvement comparing younger (<40 years), middle age (40–60 years) and older patients (≥60 years). Previous studies have shown that incidences in visual improvement do not vary significantly across age and gender in patients with uveitis [17–19], though Tranos *et al*. did report less visual improvement among elderly patients with uveitis [17]. It has been postulated that the gradual decrement of retinal cell function of aged individuals could be associated with the breakdown of the blood-retinal barrier and the inadequate ability to pump out the fluid through the retinal pigment epithelium [17, 20].

The favorable prognosis was also influenced by the anatomical type of the uveitis since an anterior location of inflammation may reflect a greater chance of sustained visual recovery; intermediate, posterior and panuveitis were predictive of a lesser incidence of sustained visual improvement. Conversely, sustained improvement in the CST was not associated with the anatomical type of uveitis. From analyzing anatomic sites of inflammation, posterior uveitis was the predominant anatomical diagnosis in our cohort, followed by anterior uveitis, panuveitis, and intermediate uveitis. Specific uveitis entities linked to the common development of UME included Birdshot chorioretinopathy and seronegative spondyloarthropathies, however 23.2% of our cohort with UME was of undetermined etiology. Interestingly, uveitis forms that are not limited to ocular involvement had a more favorable prognosis.

The therapeutical approach to inflammatory ME differs according to its laterality: in unilateral ME, local treatment modalities are preferred, while the use of systemic immunomodulatory drugs might be necessary in bilateral cases [21–22]. In our patient population, bilateral UME was associated with more favorable prognosis, however we did not find differences in the treatment modality used. This is partly because uveitis encompasses a heterogeneous group of inflammatory pathologies and frequently the treatment of UME involves the use of multiple therapeutic strategies over time.

Several clinical findings, if present during follow-up, accurately predicted sustained visual improvement, such as the presence of anterior chamber cells. Better control of anterior chamber cells was associated with higher sustained visual and CST improvement. Although the study was not designed to evaluate the various treatment strategies for ME, the benefit associated with control of inflammation suggests that treatments which successfully control inflammation are also beneficial for ME [19]. This could also explain why systemic and bilateral forms present a favorable prognosis maintained over time. The fact that most of these forms of uveitis have received systemic treatments may explain the long-term control of the inflammation since these treatments last much longer than the local modalities that require multiple injections. We recognize however the number of eyes included in the analysis limits the accuracy of an estimate and more studies are needed.

Another important finding of this study is the association between the integrity of the VMI with the macular thickness and the visual prognosis. Previous studies of alteration in the VMI and UME have also described the detrimental clinical effects of the epiretinal membrane formation as well as a higher risk of treatment failure for UME [23–24].

Our data also suggested that bigger cyst diameter in the CME pattern correlated with the prognosis, since larger sizes of the cysts were associated with less favorable prognosis. Cyst size therefore may be of value as a correlating morphological parameter with central retinal function and visual recovery [25]. Analysis of the current series did not find any other association in other patterns of UME. Lehpamer et al. reported that although SFR on OCT was associated with greater CST and worse VA at baseline, it was associated with good respond to treatment and with favorable prognosis [26]. Although previously published data comparing UME with and without SRF has shown an association of SRF with favorable prognosis [27], we have not found a significant association. This was likely because of the small sample of eyes with SRF included. We cannot comment on the prevalence of patterns of ME in specific forms of uveitis, because the number of cases from each specific form in our cohort of cases was too small.

The strengths of the study included the prospective design, use of standardized masked data collection protocols, a centralized masked reading center for the evaluation of OCT images, and recruitment from multiple uveitis referral centers. The primary limitation of this study was the number of eyes included in the analysis, which limits the precision of the estimates.

To our knowledge, this is the first prospective study that takes into account not only an anatomical improvement outcome but also a functional outcome to predict favorable prognosis of UME. The main reason for carrying out this study approach is our experience in the clinical practice where we have observed eyes with reduction in macular thickness without improving VA. These findings are not surprising as VA depends on diverse factors including media opacities, macular atrophy, macular ischemia, as well as permanent structural anomalies. For this reason, we designed a study to analyze both variables in order to predict which factors could positively influence eyes which developed well in both VA and CST.

In conclusion, our cohort of cases with UME presenting to tertiary uveitis centers lead to a reasonably favorable prognosis for sustained visual and anatomic improvement over time. Several eye-specific factors, if present at baseline, predicted favorable prognosis. Bilateral presentation of UME, systemic involvement, and cases with active anterior chamber cells, tended to show sustained improvement and favorable prognosis. In contrast, thicker CST, altered VMI or big dimension of the cystic spaces predicted less favorable visual prognosis.
